# A novel neutralizing human monoclonal antibody broadly abrogates hepatitis C virus infection *in vitro* and *in vivo*

**DOI:** 10.1016/j.antiviral.2017.10.015

**Published:** 2017-12

**Authors:** Isabelle Desombere, Ahmed Atef Mesalam, Richard A. Urbanowicz, Freya Van Houtte, Lieven Verhoye, Zhen-Yong Keck, Ali Farhoudi, Koen Vercauteren, Karin E. Weening, Thomas F. Baumert, Arvind H. Patel, Steven K.H. Foung, Jonathan Ball, Geert Leroux-Roels, Philip Meuleman

**Affiliations:** a Department of Clinical Chemistry, Microbiology and Immunology, Ghent University, Ghent, Belgium; b Therapeutic Chemistry Department, National Research Centre (NRC), Dokki, Cairo, Egypt; c School of Life Sciences, The University of Nottingham, Nottingham, NG7 2RD, UK; d Nottingham Digestive Diseases Centre, National Institute for Health Research (NIHR) Nottingham Biomedical Research Centre, Nottingham University Hospitals NHS Trust and The University of Nottingham, Nottingham, NG7 2UH, UK; e Department of Pathology, Stanford University School of Medicine, Stanford, CA, USA; f Inserm U1110, Institut de Recherche sur les Maladies Virales et Hépatiques, Strasbourg, France; g Université de Strasbourg, Strasbourg et Pole Hépato-digestif, Hôpitaux Universitaires de Strasbourg, Strasbourg, France; h MRC-University of Glasgow Centre for Virus Research, University of Glasgow, Glasgow, UK

**Keywords:** Hepatitis C virus, Envelope protein, Neutralizing antibody, Chimeric mice, Liver transplantation, Vaccine

## Abstract

Infections with hepatitis C virus (HCV) represent a worldwide health burden and a prophylactic vaccine is still not available. Liver transplantation (LT) is often the only option for patients with HCV-induced end-stage liver disease. However, immediately after transplantation, the liver graft becomes infected by circulating virus, resulting in accelerated progression of liver disease. Although the efficacy of HCV treatment using direct-acting antivirals has improved significantly, immune compromised LT-patients and patients with advanced liver disease remain difficult to treat. As an alternative approach, interfering with viral entry could prevent infection of the donor liver. We generated a human monoclonal antibody (mAb), designated 2A5, which targets the HCV envelope. The neutralizing activity of mAb 2A5 was assessed using multiple prototype and patient-derived HCV pseudoparticles (HCVpp), cell culture produced HCV (HCVcc), and a human-liver chimeric mouse model. Neutralization levels observed for mAb 2A5 were generally high and mostly superior to those obtained with AP33, a well-characterized HCV-neutralizing monoclonal antibody. Using humanized mice, complete protection was observed after genotype 1a and 4a HCV challenge, while only partial protection was achieved using gt1b and 6a isolates. Epitope mapping revealed that mAb 2A5 binding is conformation-dependent and identified the E2-region spanning amino acids 434 to 446 (epitope II) as the predominant contact domain. *Conclusion*: mAb 2A5 shows potent anti-HCV neutralizing activity both *in vitro* and *in vivo* and could hence represent a valuable candidate to prevent HCV recurrence in LT-patients. In addition, the detailed identification of the neutralizing epitope can be applied for the design of prophylactic HCV vaccines.

## Highlights

• Development of a novel human monoclonal antibody (mAb), designated 2A5, that targets the HCV envelope glycoprotein E2.
• mAb 2A5 efficiently neutralizes HCVpp and HCVcc in a pan-genotypic manner.
• mAb 2A5 protects human-liver chimeric mice from HCV challenge.
• Our novel mAb could be used to protect liver transplant patients from HCV recurrence.
• The 2A5 epitope represents a valuable target for the development of HCV vaccines with broad-spectrum activity.

## 1 Introduction

Approximately 130–170 million people are chronically infected with hepatitis C virus (HCV) worldwide. HCV infection represents a major health problem since more than 70% of infected individuals develop chronic viral hepatitis that can ultimately progresses to liver cirrhosis and hepatocellular carcinoma (HCC). While a prophylactic vaccine is still lacking, the landscape of HCV treatment has been revolutionized by the approval of multiple new generation protease, NS5A and polymerase inhibitors. Nevertheless, certain patient populations, such as liver transplant recipients, patients with advanced liver disease and genotype (gt) 3 infected individuals remain difficult to treat (Zeuzem et al., 2014). Moreover, recent evidence suggests that direct-acting antivirals (DAA)-induced cure does not eliminate the risk of hepatocellular carcinoma, in particular in patients with fibrosis, and even may be associated with HCC recurrence early after HCV clearance (Baumert et al., 2017, Conti et al., 2016, Llovet and Villanueva, 2016, Reig et al., 2016). Upon liver transplantation (LT), re-infection of the liver graft by circulating virus is unavoidable and viral variants with resistance-associated substitutions have been identified in patients that failed DAA-based therapy. Furthermore, DAA treatment in solid organ transplantation can result in severe adverse effects (Hogan et al., 2017).

Besides its pivotal role in viral entry, the E2 envelope protein represents the main target for the host's adaptive immune system and the induction of neutralizing antibodies (nAbs). The study of HCV has long been hampered by the lack of robust *in vitro* and *in vivo* models. Retroviral particles pseudotyped with HCV envelope glycoproteins (HCVpp) and the HCV cell culture system (HCVcc) have proven very valuable to study virus binding and entry (Catanese and Dorner, 2015). On the other hand, mice with chimeric humanized liver currently represent the most reliable *in vivo* animal model alternative to the chimpanzee for the study of HCV (Mercer et al., 2001, Meuleman et al., 2005). We and others have used this model extensively to study HCV biology, to determine the neutralizing capacity of monoclonal and polyclonal antibodies that target the virus or one of its receptors, and for the evaluation of novel therapeutic approaches (Mesalam et al., 2016).

The impact of cell-mediated immunity on HCV clearance has been shown in multiple studies (Heim and Thimme, 2014, Park and Rehermann, 2014), whereas it remains less clear to what extent the humoral immune response plays a role. Nevertheless, spontaneous recovery from HCV infection seems to be associated with the early development of broadly neutralizing antibodies (Ndongo et al., 2010, Osburn et al., 2014) and highly neutralizing antibodies have been detected in injection drug users that tested HCV RNA negative (Swann et al., 2016b). In addition, high levels of broad neutralizing antibodies have been associated with reduced disease severity, lower viral loads and higher SVR rates after pegylated-interferon/ribavirin therapy (Hamed et al., 2008, Ndongo et al., 2010, Swann et al., 2016a). Furthermore, weak or absent antibody neutralization in HCV/HIV co-infected and agammaglobulinemia patients respectively, correlate with the severity of liver disease and lower diversity within the HCV quasispecies pool (Booth et al., 1998, Maurin et al., 2015). Altogether, these findings reflect the importance of the humoral immune response during HCV infection.

Interference with HCV entry by passive immunization or by vaccine-induced nAbs represents an attractive approach for the prevention of HCV infection, especially in chronically infected patients undergoing liver transplantation (Felmlee et al., 2016). Polyclonal antibodies from HCV infected patients as well as monoclonal antibodies (mAbs) were able to protect chimpanzees and humanized mice from experimental HCV infection (Bukh et al., 2015, Desombere et al., 2016, Eren et al., 2006, Law et al., 2008, Meuleman et al., 2011, Morin et al., 2012, O'Shea et al., 2016, Vanwolleghem et al., 2008). In addition, mAb MBL-HCV1 significantly delayed viral rebound following liver transplantation, while complete protection was reported when combined with DAAs (Chung et al., 2013, Smith et al., 2017). The limitation of this antibody is however that it is associated with rapid viral escape without compromising viral fitness. In the present study, we describe the development and *in vitro* and *in vivo* characterization of a novel human monoclonal antibody (2A5) targeting the HCV envelope.

## 2 Materials and methods

(A more detailed description of all material and methods can be found in an online supplement).

### 2.1 Generation of mAb 2A5

Hybridomas producing mAb 2A5 directed to the envelope of HCV were generated as described before (Depraetere et al., 2001). In brief, human peripheral blood mononuclear cells (PBMC) collected from an individual chronically infected with HCV of gt1b were injected in the spleen of two optimally conditioned NOD-SCID mice (originally purchased from Charles River) (1 × 10^7^ cells per animal). Six days later the mice were bled and their plasma anti-E1E2 antibody titer was measured using an in-house immunoassay (EIA). On day 7, the mouse displaying the highest anti-E1E2 titer was sacrificed and a cell suspension of the spleen was prepared that was then mixed with K6H5/B5 heteromyeloma cells (kindly provided by Dr. Kris Thielemans, Free University of Brussels (VUB), Brussels, Belgium) at a 4:1 ratio. Polyethylene glycol 1500 (50% v/v; Boehringer Mannheim, Mannheim, Germany) was added for 2 min and then washed away. Fused cells (5 × 10^4^) were cultured in microtiter plates in 200 μL of medium supplemented with human recombinant insulin (10 μg/mL, Boehringer Mannheim), ouabain (1 μM, Sigma, St. Louis, MO), hypoxanthine-aminopterin-thymidine (Life Technologies, Belgium) and 10% v/v BM Condimed HI (Boehringer Mannheim). Cultures were replenished with fresh medium every other day and individual wells were checked for cell growth first and anti-E1E2 IgG production subsequently. Eight anti-E1E2 IgG-producing cultures were selected, subcloned several times and further expanded. After initial screening for reactivity against the HCV E1E2 protein (EIA), the neutralizing potential of the supernatant of all growing cultures was tested with neutralization assays using HCV pseudoparticles (HCVpp) of gt1a (isolate H77c). The human mAb with the strongest neutralizing capacity was selected and designated 2A5. Hybridoma cells producing mAb were propagated in a two-compartment bioreactor (Integra) and the antibody-containing culture supernatant was changed weekly. mAb 2A5 was purified using a protein G column (GE Healthcare Life Sciences) and concentrated using Amicon Centrifugal filters (Merck Millipore). The mAb 2A5 content of this preparation was determined using a human IgG ELISA Quantitation Set (Bethyl Laboratories).

### 2.2 *In vitro* experiments

The binding affinity of mAb 2A5 was tested using an enzyme immunoassay (EIA) with cell lysates containing recombinant HCV E1E2 glycoproteins as previously described (Owsianka et al., 2005). The neutralization potential of this mAb was also tested using HCVpp and HCVcc systems. A detailed description of cells, antibodies and methods used in all *in vitro* experiments can be found in the online supplement.

### 2.3 *In vivo* HCV challenge

Human liver chimeric mice were produced as previously described (Mercer et al., 2001, Meuleman et al., 2005). Briefly, two weeks after birth, homozygous uPA^+/+^-SCID mice were transplanted by intra-splenic injection with 10^6^ cryopreserved primary human hepatocytes (donor HH223; BD Biosciences, Belgium). In passive immunization studies, mice were intraperitoneally injected with 1 mg of mAb 2A5 three days before challenge with a 100% infectious dose of gt1a (mH77; 10^4^ IU), gt1b (mP05; 10^4^ IU), gt4a (mED43; 10^4^ IU) or gt6a (mHK6a; 10^5^ IU) (Desombere et al., 2016, Meuleman et al., 2011). Viremia was quantified using the COBAS Ampliprep/COBAS TaqMan HCV test (Roche Diagnostics). The limit of quantification (LOQ) in diluted plasma was 750 IU/mL. The study protocol was approved by the local animal ethics committee.

### 2.4 Statistical analysis

Statistical significance was calculated by Wilcoxon's matched-pairs signed-ranks test using GraphPad Prism software version 6. P-values below 0.05 were considered statistically significant.

## 3 Results

### 3.1 mAb 2A5 efficiently binds and neutralizes most prototype and patient-derived HCV strains

We compared the binding and neutralizing activity of mAb 2A5 with the broadly neutralizing anti-E2 mAb AP33, using the envelopes of infectious HCV variants isolated from: (i) prototype HCV-strains and (ii) gt1b patient-derived strains which were selected during transmission to humanized mice. The relative affinity of mAbs 2A5 and AP33 for E1E2 derived from prototype HCV gt1a (H77c), 2a (JFH1) and 3a (S52) and for chronic patient isolates P09_VA, P12_VA and P12_1091 was determined using GNA-capture ELISA (Fig. 1a). Dose-response experiments demonstrate that mAb 2A5 usually performs better than mAb AP33 (P = 0.031). The apparent affinity depends on the isolate with half maximal effective concentration (EC_50_) values ranging between 0.109 and 1.215 μg/mL (Fig. 1b).

**Fig. 1:**
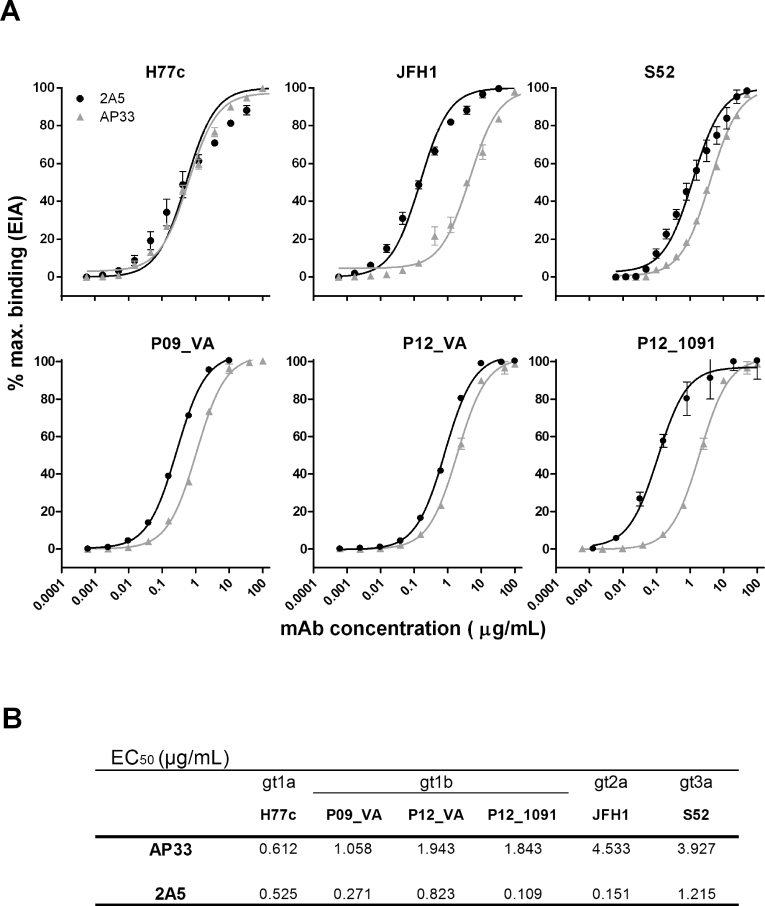
**Binding characteristics of mAbs 2A5 and AP33. (A)** mAbs 2A5 and AP33 were serially diluted and incubated on plates pre-coated with cell lysates containing HCV E1E2 derived from prototype isolates (H77c, JFH1, S52) and gt1b patient-derived viral isolates (P09_VA, P12_VA, P12_1091). Dose-dependent binding is expressed as percentage of maximal binding (mean ± standard deviation (error bars)). **(B)** EC_50_ values were calculated from dose-response curves shown in **(A)**. All conditions were performed in duplicate.

The neutralization potential of mAb 2A5 was first investigated using HCVpp expressing envelope glycoproteins from 3 prototype viruses (H77c, JFH1 and S52) and 5 patient-derived gt1b viral variants (P09_VA, P09_VB, P09_779, P12_VA and P12_1091). Viral entry was inhibited in a dose-dependent manner and for most viral strains the neutralizing potential of mAb 2A5 was superior to that of AP33 (P = 0.25) (Fig. 2a). Similar to the E1E2-binding data, viral strain S52 (gt3a) was difficult to neutralize and viral strain P12_1091 (gt1b) was very efficiently neutralized (Fig. 2b). To extend these observations and to elaborate on the cross-neutralizing potential of mAb 2A5, HCVpp neutralization experiments were performed using HCV envelope glycoproteins derived from clinical samples covering additional genotypes/strains (gt1b (UKN1B5.23), gt2a (UKN2A1.2 and J6), gt2b (UKN2B2.8), gt3a (UKN3A13.6), gt4 (UKN4.11.1), gt5 (UKN5.15.7) and gt6 (UKN6.5.8)). The obtained results demonstrate that neutralization by mAb 2A5 is mainly strain dependent and that strains J6 and UKN4.11.1 can hardly be neutralized (Table S1).

**Fig. 2:**
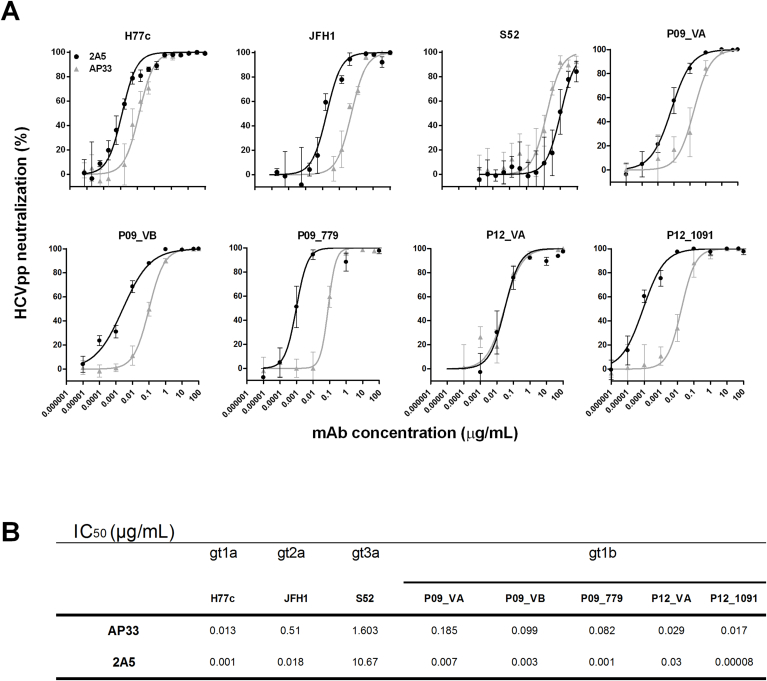
**Neutralization of HCVpp by mAbs 2A5 and AP33. (A)** HCVpp expressing E1E2 derived from prototype isolates (H77c, JFH1, S52) and gt1b patient-derived viral isolates (P09_VA, P09_VB, P09_779, P12_VA and P12_1091) were incubated for 1 h at 37 °C with serial dilutions of mAbs 2A5 or AP33 and added to Hep3B cells. HCVpp entry was analyzed by luciferase reporter gene expression and normalized to isotype controls. Neutralization is expressed as % neutralization (mean ± standard deviation (error bars)). **(B)** IC_50_ values were calculated from dose-response curves shown in **(A)**. All conditions were performed in quadruplicate.

**Fig. 3:**
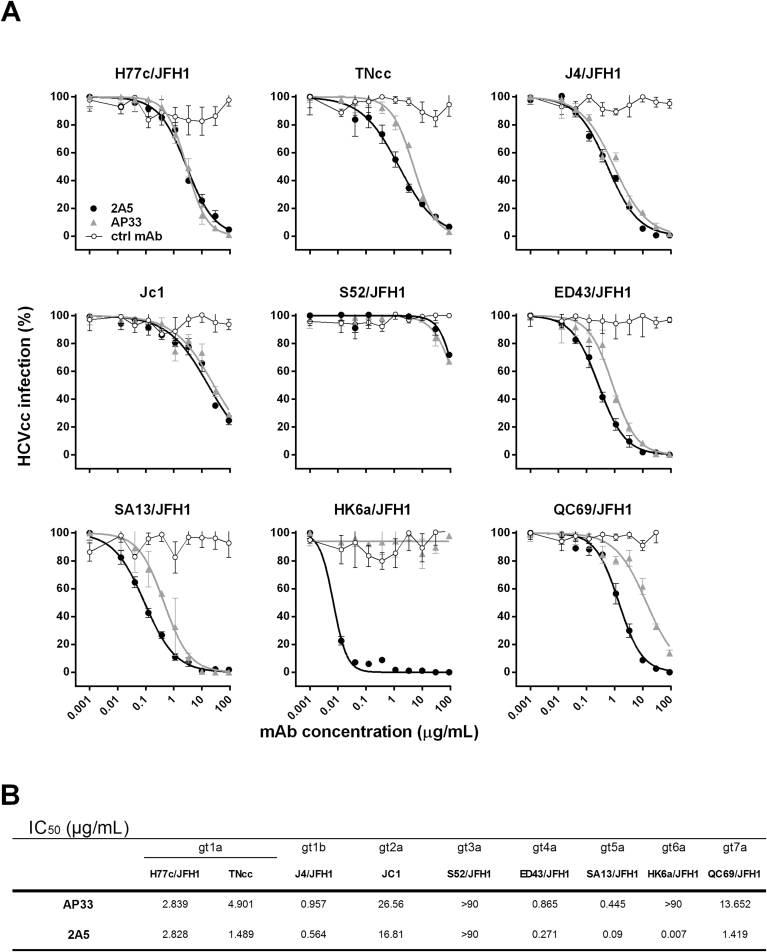
**Neutralization of HCVcc by mAbs 2A5 and AP33. (A)** HCVcc expressing the structural proteins of genotype 1a, 1b, 2a, 3a, 4a, 5a, 6a and 7a isolates were pre-incubated with three-fold serial dilutions of mAbs 2A5, AP33 or a control Ab. The mixture was transferred to Huh7.5.RFP cells and incubated for 4 h before washing. Two days later, HCV-infected foci were visualized using an NS5A-specific antibody and counted. Results are expressed as percentage of infectivity (mean ± standard deviation (error bars)). **(B)** IC_50_ values were calculated from dose-response curves shown in **(A)**. All conditions were performed in triplicate.

To further corroborate the neutralizing potency of mAb 2A5 we utilized the HCVcc system covering multiple HCV strains: gt1a (H77c/JFH1 and full length TNcc), gt1b (J4/JFH1), gt2a (JC1), gt3a (S52/JFH1; UKN3A1.28c; and UKN3A13.15), gt4a (ED43/JFH1), gt5a (SA13/JFH1), gt6a (HK6a/JFH1) and gt7a (QC69/JFH1) (Fig. 3a and Fig. S1). Overall, results demonstrate that mAb 2A5 efficiently neutralizes most HCV strains, with half maximal inhibitory concentration (IC_50_) values comparable or superior to the neutralization observed with AP33 (P = 0.0078). The gt6a strain HK6a/JFH1, which could not be neutralized by AP33, was very efficiently neutralized by 2A5 (IC_50_ = 0.007 μg/mL). Strain S52/JFH1 was barely neutralized, in line with the E1E2 binding and HCVpp neutralization results (Fig. 3b).

### 3.2 Protection by mAb 2A5 from HCV challenge *in vivo*

**Fig. 4:**
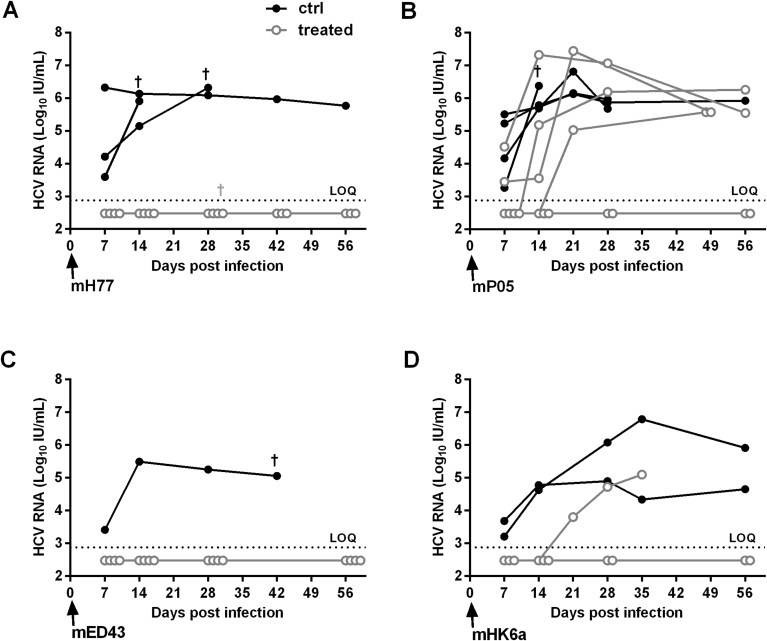
**mAb 2A5 protects human liver chimeric mice from HCV challenge.** Three days before viral infection, humanized mice were passively immunized with 1 mg of mAb 2A5. Mice were challenged with mH77 (gt1a, 10^4^ IU HCV RNA), mP05 (gt1b, 10^4^ IU), mED43 (gt4a, 10^4^ IU) or mHK6a (gt6a, 10^5^ IU). Viremia was quantified until week 8 post infection. 2A5-treated and control mice are represented by closed and open circles, respectively. Mice that scored HCV RNA negative are displayed below the dashed line representing the limit of quantification (LOQ; HCV RNA <750 IU/mL).

The neutralizing potency of mAb 2A5 was also tested *in vivo.* Human liver chimeric uPA-SCID mice were passively immunized with mAb 2A5 or left untreated (control) and challenged three days later with HCV of gt1a (mH77c), gt1b (mP05), gt4a (mED43) and gt6a (mHK6a) (Fig. 4). After challenge with mH77c, all four 2A5-treated mice remained HCV RNA negative throughout the 8-week observation period, whereas the 3 control mice became viremic within the first week after virus injection. Similarly, challenge with mED43 resulted in complete protection of all 2A5-treated mice. After challenge with mHK6a, all 2A5-treated mice remained HCV RNA negative until the second week after challenge. At week 3, HCV RNA became detectable in one 2A5-treated mouse (6.35 × 10^3^ IU/mL), while the others remained undetectable until week 8. Again, HCV RNA was readily detected in the control mice from week 1 onwards. Challenge with the difficult-to-neutralize mP05 isolate (Fafi-Kremer et al., 2010, Fofana et al., 2012) ultimately resulted in protection in 2 out of 6 treated mice.

Although the human IgG plasma levels in 2A5-treated mice were very variable (measured at week 1, ranging from 23 to 202 μg/mL), no correlation could be found between the circulating mAb levels and the viral kinetics (Table S2). To test the presence of resistant variants in non-protected mice (gt1b and gt6a), we amplified the E1E2 region of the virus present in the plasma from treated and control mice. Sequence analysis showed comparable viral quasispecies variability in the E2 region between treated and control groups, and no resistant mutants could be identified (results not shown).

### 3.3 mAb 2A5 mainly recognizes the E2-region spanning amino acids 434 to 446

In order to delineate the epitope that is targeted by mAb 2A5 we initially scanned the complete E1E2 sequence using a custom-made peptide microarray (PEPperCHIP^®^, PEPperPRINT GmbH, Germany) wherein the complete E1E2-sequence, originating from a dominant sequence within the quasispecies of the patient from whom mAb 2A5 was generated, is displayed as 15-mer peptides with an overlap of 14 AA. The peptide sequences and complete chip layout are detailed in supplementary File S1. Using this microarray platform, the E2-region AA434-443 was identified as a major binding region for mAb 2A5 (Fig. S2).

**Fig. 5:**
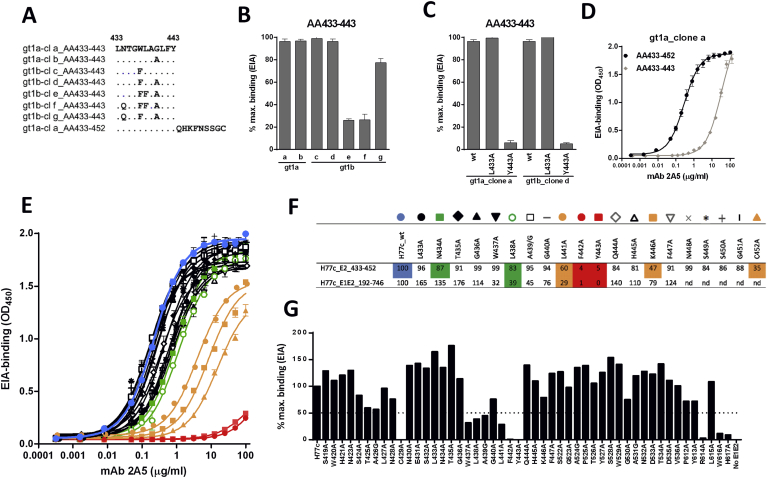
**Epitope mapping of mAb 2A5.** The binding pattern of mAb 2A5 was analyzed using gt1a- and gt1b-derived sequences, of which the E2 region spanning amino acids (AA)433–443 of the different clones (cl) is shown **(A)**. After incubation with surface-attached peptides, bound antibodies were detected with an HRP-conjugated anti-human secondary antibody. The 2A5-binding pattern was analyzed using mAb (50 μg/mL) and peptides spanning AA-region 433–443, derived from naturally occurring gt1a (clone a-b) and gt1b (clone c-d) isolates **(B)**, and A-substituted peptides (L433A, Y443A) from gt1a_clone a and gt1b_clone d **(C)**. Binding was normalized to the best binding peptide (100% binding). **(D)** To elaborate on the length of the epitope, the efficiency of 2A5-binding was compared between peptides covering region AA433-443 and peptides including an additional upstream sequence (AA433-452), all derived from gt1a_clone a. **(E)** To further elaborate on 2A5-anchor residues within the region of interest (AA433-452), the binding efficiency was compared between peptides derived from the prototype isolate H77c and their A-substituted counterparts. The corresponding color-codes, representing the different A-substituted sequences, and the % binding relative to native H77c at 10 μg mAb/mL are shown in **(F)**. **(G)** To confirm these results and to reveal additional contact residues for mAb binding, epitope mapping was done using E1E2 proteins of the H77c isolate with A-substitutions in 3 regions (AA419-447, AA522-536 and AA612-617). 2A5-binding to full-length native and mutated E1E2 cell lysates was analyzed. Results represent the % binding relative to E1E2_H77c at 10 μg mAb/mL **(F, G)**.

The involvement of this region in 2A5-binding was confirmed and further scrutinized using an in-house enzyme EIA with peptides spanning AA433-443 derived from different gt1a and gt1b natural viral isolates (AA sequence see Fig. 5a). From these binding experiments we could conclude that 2A5-binding is unchanged when (i) position AA440 is occupied by amino acid G or A, or (ii) position AA437 is occupied by amino acid W or F. At position AA434, N confers better binding than Q and at position AA438, L is much more favorable for binding than F (Fig. 5b). To elaborate on the anchor residues, mutant peptides were generated wherein residues at position AA433 and AA443 were replaced by alanine (L433A and Y443A) (Fig. 5c). Results demonstrate that position 443 plays a critical role in optimal binding. In addition, comparison of 2A5-binding to the peptide AA433-443 with that to a peptide covering the region AA433-452, showed enhanced affinity towards the longer peptide indicating additional contact residues within the region adjacent and downstream from AA443 (Fig. 5d). To further elaborate on 2A5-anchor residues within region AA433-452, the binding efficiency was compared between the peptide representing the prototype isolate H77c and its alanine-substituted counterparts (Fig. 5e and f). Our data indicate that AA434, AA438, AA441, AA442, AA443 and AA446 are important for 2A5 binding, with AA442 and AA443 being major anchor residues.

To confirm these results, our peptide binding assays were repeated in the context of full-length E1E2 protein. 2A5-binding was tested for binding to wild-type H77c E1E2 protein and its mutants in which the AA spanning regions 419–447, 522–536 and 612–617 were alanine-substituted (Fig. 5g). Based on the reduction in mAb binding, our analyses revealed several regions within E2 that are critical for 2A5-binding: AA424-428, AA437-443, AA446, AA530 and AA612-617. Results obtained for AA437-446 are largely comparable with EIA-peptide data and are shown in Fig. 5f. The finding that several non-adjacent regions within E2 are involved in binding and that mutation at position C429 (forming a disulfide bond) completely abrogates binding, suggests that mAb 2A5 recognizes a conformational epitope.

**Fig. 6:**
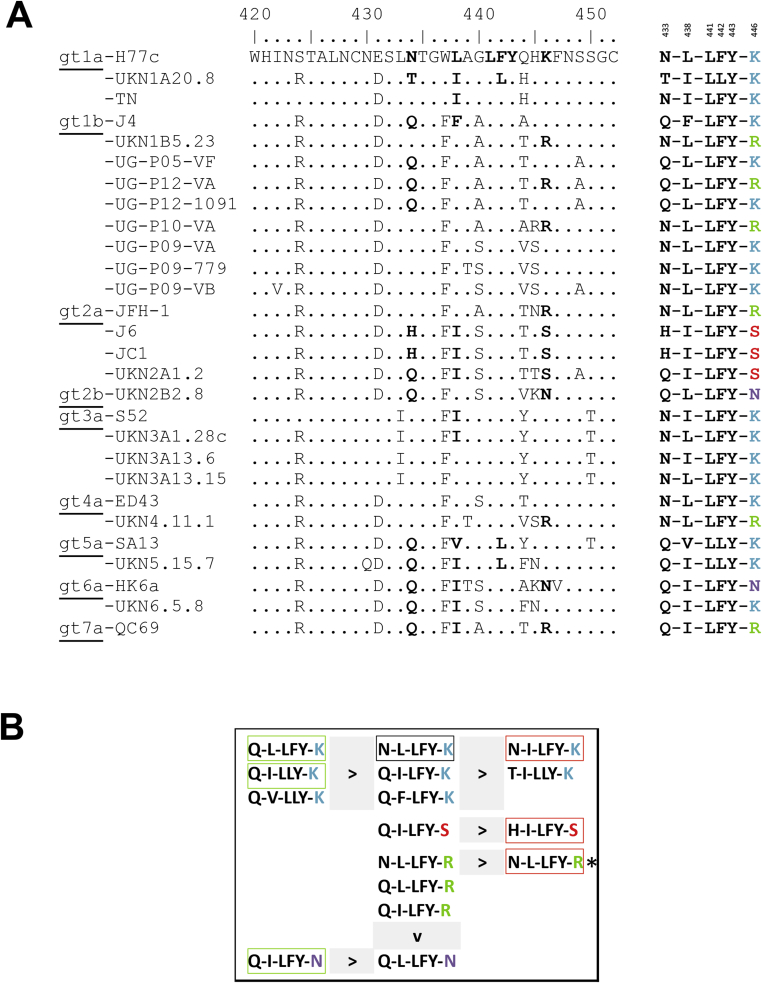
**Main binding motif recognized by mAb 2A5. (A)** Alignment of the E2-region spanning AA420-452 of the different HCV strains used in the binding and neutralization studies. Residues found critical for mAb 2A5-binding (AA433, AA438, AA441, AA442, AA443 and AA446) are highlighted and, for each strain, the simplified AA-motif is shown on the right. **(B)** A hierarchy in neutralization potential was deduced for the binding motifs. The AA-motif corresponding to the H77c strain is marked with a black box. AA-motifs shown on the left-side of ‘>’ have better neutralization properties compared to AA-motifs shown on the right-side. Motifs from ‘difficult to neutralize’ strains HCV-S52, JC1 and J6 are highlighted in red boxes. Motifs from HCV-strains that can be efficiently neutralized by 2A5 (P12_1091, HK6a and UKN5.15.7) are indicated in green boxes. (*) represents **i**dentical motifs with different neutralization efficiencies.

Alignment of the E2-region spanning AA420-452 of the different HCV strains used in the binding and neutralization studies is shown in Fig. 6a. Residues found critical for 2A5-binding in that region are highlighted and, for each strain, the simplified AA-motif is shown on the right. Based on aforementioned experiments, a neutralization-hierarchy is deduced for the different motifs that, for most motifs, can largely predict the neutralization outcome (Fig. 6b). However, two motifs are linked to opposing neutralization data: (i) motif *N-I-LFY-K* is present in strains TNcc (IC_50_: 1.49 μg/mL) and S52 (IC_50_: >90 μg/mL), and (ii) motif *N-L-LFY-R* is present in strains JFH1 (IC_50_: 0.02 μg/mL), UKN1B5.23 (IC_50_: 0.57 μg/mL) and UKN4.11.1 (IC_50_: 38 μg/mL). Furthermore, the motif *Q-L-LFY-K*, which was very efficiently neutralized *in vitro*, could barely be neutralized *in vivo* (mP05).

### 3.4 The epitope recognized by mAb 2A5 is conformational

**Fig. 7:**
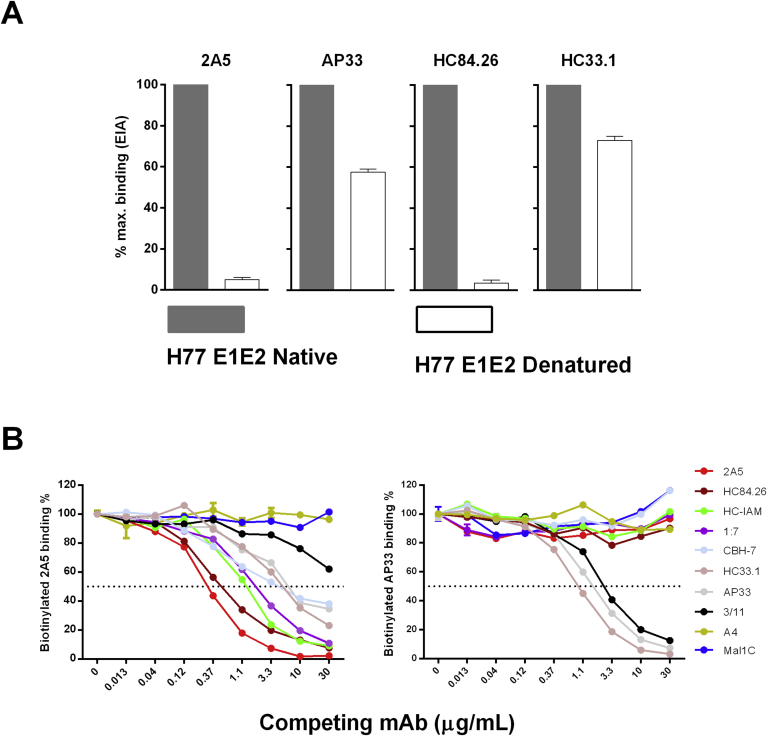
**Recognition by mAb 2A5 is conformation dependent. (A)** To reveal whether mAb 2A5 recognizes a linear or a conformational epitope, mAbs 2A5, AP33, HC84.26 or HC33.1 were incubated at 5 μg/mL with native as well as denatured full-length H77c E1E2 cell lysates. Bound antibodies were detected with HRP-conjugated secondary antibodies and the signal was normalized to that obtained with the native protein. **(B)** To analyze whether 2A5 competes with other mAb, a competition EIA was performed wherein anti-E2 (AP33, HC33.1, HC84.26, HC-1AM, CBH-7, 1:7 and 3/11) and anti-E1 (A4) mAbs were used as competing antibodies for binding to the E1E2 protein of the H77c isolate. Three-fold serial dilutions of competing mAbs were used starting at 30 μg/mL. The binding of biotinylated versions of mAb 2A5 (left panel) and AP33 (right panel) was measured using HRP-conjugated streptavidin. Mal1C is an irrelevant antibody that was included as negative control. All conditions were performed in duplicate.

To investigate whether the epitope recognized by mAb 2A5 is linear or conformational, binding to native and denatured H77c E1E2 protein was compared. mAbs recognizing conformational (mAb HC84.26) or linear (HC33.1 and AP33) epitopes were included for comparison (Fig. 7a). The EIA-results showed complete loss of 2A5-binding to denatured E1E2 proteins while mAbs HC33.1 and AP33 retained more than 50% of their binding activity. As expected mAb HC84.26 did not recognize denatured HCV E1E2 proteins. These results clearly demonstrate the conformation-dependency of 2A5 binding.

To analyze whether competition occurs between mAb 2A5 and other known mAbs for binding to the HCV envelope, a competition EIA was set-up wherein anti-E2 (AP33, HC33.1, HC84.26, HC-1AM, CBH-7, 1:7 and 3/11) and anti-E1 (A4) mAbs were used as competing antibodies for 2A5-binding to E1E2 of isolate H77c (Fig. 7b). Our results demonstrate that, apart from the anti-E1 mAb, all mAbs compete for 2A5-binding. As expected, only mAb 3/11 and HC33.1 compete for AP33-binding. These mAbs were previously shown to recognize similar epitopes.

## 4 Discussion

The HCV envelope proteins contain different immunogenic regions, but not all of these are suitable for vaccine design. The HVR1 region is highly immunogenic, but is known for its high mutation rate that enables viral escape; and antibodies directed to this region may interfere with broad-neutralizing ones (Keck et al., 2016a, Prentoe et al., 2016). Likewise, the immunogenic E2-epitope domain A induces antibodies with non-neutralizing activity (Keck et al., 2007). Recently it was shown that E2 protein lacking the three variable regions (HVR1, HVR2 and igVR; Δ123) induced antibodies that cross-neutralized HCV *in vitro* (Vietheer et al., 2017). During the last decade, several mAbs that target different E2-epitopes have been developed and the concept of passive immunization for protection from HCV challenge has been investigated in different studies. Treatment of chimpanzees with human mAb MBL-HCV1 protected from challenge with gt1a HCV (Morin et al., 2012). In another study, the same mAb showed a delay of HCV recurrence in patients after liver transplantation (Chung et al., 2013). In a most recent proof-of-concept study involving liver transplantation patients, combination therapy of sofosbuvir and mAb MBL-HCV1 completely protected from HCV re-infection (Smith et al., 2017). The murine mAb AP33, targeting a conserved region within E2, has been extensively studied and shows broad cross-neutralizing activity to most HCV genotypes (Desombere et al., 2016, Owsianka et al., 2005, Tarr et al., 2006, Urbanowicz et al., 2015).

In the present study, we generated a human mAb, designated 2A5, from a gt1b chronic HCV patient. We demonstrate that mAb 2A5 efficiently binds and neutralizes most HCV strains, with EC_50_ and IC_50_ values comparable or lower than those of AP33. However, the gt6a strain HK6a/JFH1, which was insensitive to AP33, was very efficiently neutralized by 2A5. This chimeric HK6a/JFH1 virus contains cell culture adaptive mutations at multiple sites including the region encompassing the epitope critical for AP33 binding, which might explain its resistance to AP33 neutralization (Gottwein et al., 2009). The gt3a chimeric HCVcc S52/JFH1 was barely neutralized, confirming the results obtained with the HCVpp system. Remarkably, mAb 2A5 was able to neutralize two other gt3a chimeric viruses (UKN3A1.28c/JFH1 and UKN3A13.15/JFH1), supporting the concept that neutralization sensitivity is determined at strain rather than genotype level (Tarr et al., 2011, Urbanowicz et al., 2015). Despite the sometimes relatively high coefficient of variation, which is inherent to the neutralization assays used, our data indicates that mAb 2A5 seems to be the most broadly neutralizing antibody identified so far. In contrast to most recently published studies that use ‘prototype’ strains for neutralization assays and animal challenge (Akazawa et al., 2013, Bukh et al., 2015, Xiao et al., 2015), we also included patient-derived viral variants. These variants probably better reflect the diversity of natural HCV infection and are therefore an important tool for the evaluation of HCV-specific mAbs.

During the last decade, the human liver chimeric mouse model has proven to be very valuable as an alternative to the chimpanzee for the *in vivo* study of HCV (Mesalam et al., 2016). Using this small animal model, we previously reported that passive immunization with polyclonal antibodies targeting the HCV envelope could protect from HCV infection (Meuleman et al., 2011, Vanwolleghem et al., 2008). We now utilized this model to evaluate whether mAb 2A5 can confer protection from viral challenge with different HCV isolates. A single dose of 1 mg of mAb 2A5 was able to confer protection against mH77c (gt1a), mED43 (gt4a) and HK6a (gt6a) challenge. Since control mice became HCV RNA positive early after infection, the protection can clearly be attributed to the presence of mAb 2A5 in circulation. This *in vivo* result correlates with the observed *in vitro* neutralization data. The efficacy of mAb 2A5 was also tested against ‘difficult-to-neutralize’ strains. For this, we used a gt1b viral escape isolate, obtained from a liver-transplanted patient (mP05) (Fafi-Kremer et al., 2010). After mP05 challenge, only partial protection was found, but breakthrough mice showed delayed viral kinetics during the first week(s) post inoculation. Previously, it was demonstrated that this isolate was highly resistant to antibody neutralization and can very efficiently enter cells by optimal receptor usage (Fafi-Kremer et al., 2010, Fofana et al., 2012). The single mAb dose used in our current study corresponds to 100 mg/kg, which is lower compared to other studies using either higher and/or multiple doses (Keck et al., 2016b, Law et al., 2008, O'Shea et al., 2016).

To delineate the epitope targeted by mAb 2A5 we initially scanned the complete E1E2 sequence. Using a peptide microarray in combination with traditional peptide-EIA, we could localize the main epitope to the E2-region spanning AA434-446, also known as ‘epitope II’ (Zhang et al., 2007). This region has also been shown to be recognized by nAbs (Keck et al., 2012), as well as peptide-affinity-enriched neutralizing polyclonal sera (Tarr et al., 2012). Surprisingly and somewhat controversially, this region is also thought to be recognized by antibodies that may interfere with the neutralization mediated by antibodies recognizing the AP33-eptitope (epitope I; AA412-423) (Zhang et al., 2007). Using alanine mutants the main 2A5-anchor residues within AA433-446 were identified as AA434, AA438, AA441, AA442, AA443 and AA446; with AA442F/L and AA443Y being critical for binding. However, this does not exclude that other aromatic amino acids might be tolerated at the latter positions. We scanned this binding motif for all strains used in the study and correlated the motif with the observed neutralization. Importantly, we found identical motifs that induce opposite neutralization depending on the strain (motif *N-I-LFY-K* and motif *N-L-LFY-R)*. Furthermore, the motif *Q-L-LFY-K*, which was very efficiently neutralized *in vitro*, could barely be neutralized *in vivo* (mP05). These data reveal the importance of additional contact residues outside region AA420-452. Full-length E1E2 protein alanine scanning identified the regions AA424-428, AA530 and AA612-617 as critical for mAb 2A5 binding.

Finally, we could demonstrate that mAb 2A5 recognizes a conformational epitope, which is in line with the several non-adjacent binding regions within E2 and the abrogation of binding in the C429A protein mutant. Interestingly, 4 out of 5 E2-regions recognized by mAb 2A5, overlap with regions previously described to be important for neutralization (reviewed in (Sautto et al., 2013)): epitope II (AA434-446), domain B (AA523-540), W (AA616) and (AA698). Furthermore, the recognition of epitope II and domain B overlaps with the recognition by the mAbs AR3 (Law et al., 2008).

The strong and cross-neutralizing activity of 2A5, shown by HCVpp and HCVcc assays and confirmed *in vivo*, makes this human monoclonal antibody a valuable candidate for passive immunization, either alone or in combination with other antiviral agents, especially in patients undergoing liver transplantation. Immunoprevention of HCV infection using a broadly neutralizing antibody may be superior to post-transplant DAA treatment, especially to prevent damage to the liver graft as well as to minimize the risk of HCC and treatment-induced adverse effects. Furthermore, the identification of highly conserved residues within the E2 provides valuable information for the development of a highly efficacious, broad-spectrum prophylactic HCV vaccine.

## Figures and Tables

**figs1:**
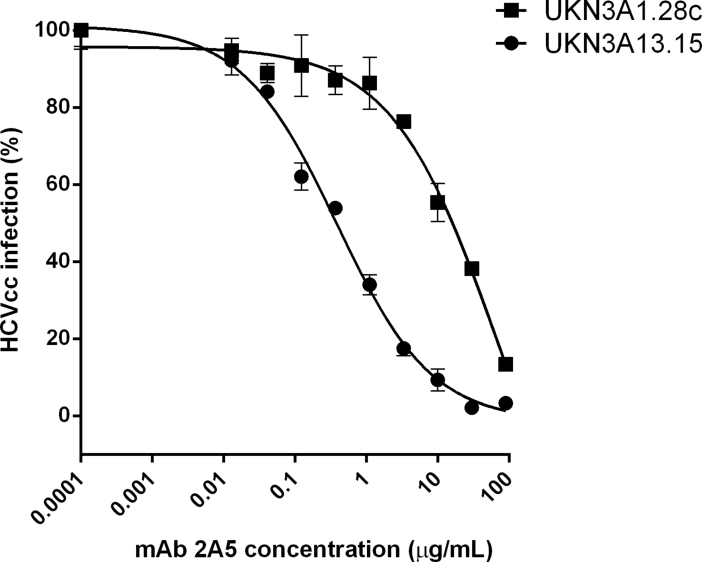


**figs2:**
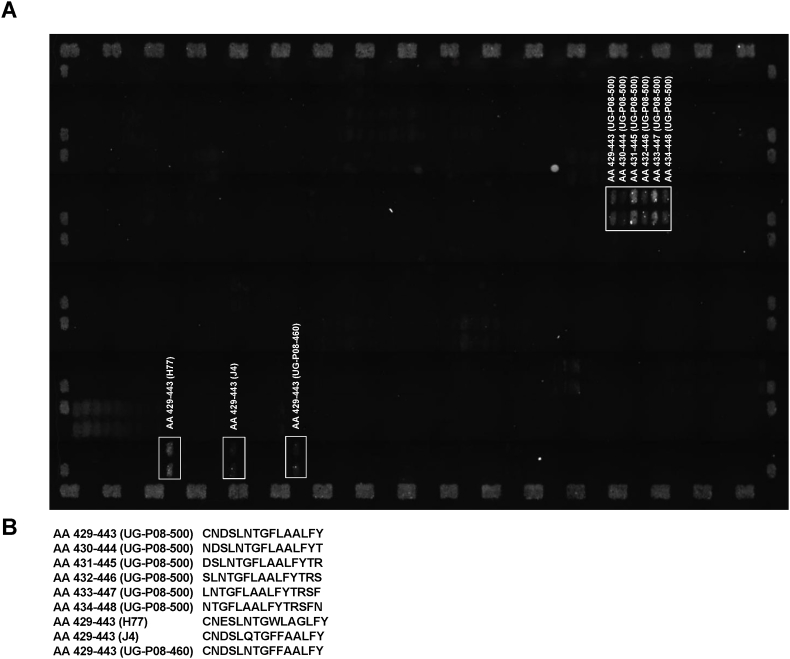


## Appendix A Supplementary data

mmc1 mmc1

mmc2 mmc2

mmc3 mmc3

mmc4 mmc4
