# Supplementary material for: A novel neutralizing human monoclonal antibody broadly abrogates hepatitis C virus infection in vitro and in vivo
Source: Antiviral Res. 2017 Dec;148:53–64. doi: 10.1016/j.antiviral.2017.10.015 (PMC5785094; doi:10.1016/j.antiviral.2017.10.015)
Supplement: mmc2 [file mmc2.docx]

**Table S1: Neutralization activity of mAb 2A5 against HCV pseudoparticles derived from clinical isolates.**

| **Isolate** | **Genotype** | **Accession no.** | **IC_50_ (µg/mL)** |
| --- | --- | --- | --- |
|  |  |  | **mAb 2A5** |
| **UKN1A20.8** | 1a | EU155192 | 7.57 |
| **UKN1B5.23** | 1b | AY734976 | 0.57 |
| **UKN2A1.2** | 2a | AY734977 | 0.47 |
| **J6** | 2a | AF177036 | 77.38 |
| **UKN2B2.8** | 2b | AY734983 | 8.78 |
| **UKN3A13.6** | 3a | AY894683 | 0.35 |
| **UKN4.11.1** | 4 | AY734986 | 38.16 |
| **UKN5.15.7** | 5 | EF427672 | 0.02 |
| **UKN6.5.8** | 6 | EF427671 | 0.34 |
