# Supplementary material for: A novel neutralizing human monoclonal antibody broadly abrogates hepatitis C virus infection in vitro and in vivo
Source: Antiviral Res. 2017 Dec;148:53–64. doi: 10.1016/j.antiviral.2017.10.015 (PMC5785094; doi:10.1016/j.antiviral.2017.10.015)
Supplement: mmc4 [file mmc4.doc]

**A novel neutralizing human monoclonal antibody broadly abrogates hepatitis C virus infection *in vitro* and *in vivo***

Isabelle Desombere^a,1,*^, Ahmed Atef Mesalam^a,b,*^, Richard A. Urbanowicz^c,d^, Freya Van Houtte^a^, Lieven Verhoye^a^, Zhen-Yong Keck^e^, Ali Farhoudi^a^, Koen Vercauteren^a^, Karin E. Weening^a^, Thomas F. Baumert^f,g^, Arvind H. Patel^h^, Steven K. H. Foung^e^, Jonathan Ball^c,d^, Geert Leroux-Roels^a^, Philip Meuleman^a, §^

**Materials and Methods**

**Cell-lines and monoclonal antibodies**

The human hepatoma cell lines (Hep3B (ATCC HB-8064) and Huh-7.5-RFP-NLS-IPS (provided by Dr. Charles M. Rice, Rockefeller University, NYC, USA)) and human embryonic kidney cell line 293T (provided by Dr. Jane McKeating, University of Birmingham, UK) were grown in Dulbecco’s Modified Eagle’s Medium (DMEM) supplemented with 10% heat-inactivated fetal calf serum (FCS), 1% non-essential AA and 1% L-glutamine. The generation of the human anti-E2 mAbs (HC84.26, HC33.1, HC-1AM, CBH-7, 1:7), the murine anti-E2 mAb (AP33), the rat anti-E2 mAb (3/11), the murine anti-E1 mAb (A4) and the human control mAb (Mal1C, anti-CSP (Plasmodium falciparum circumsporozoite protein)) has been previously described .

**Generation of mAb 2A5**

Hybridomas producing mAb 2A5 directed to the envelope of HCV were generated as described before . In brief, human peripheral blood mononuclear cells (PBMC) collected from an individual chronically infected with HCV of gt1b were injected in the spleen of two optimally conditioned NOD-SCID mice (originally purchased from Charles River) (1x10^7^ cells per animal). Six days later the mice were bled and their plasma anti-E1E2 antibody titer was measured using in-house immunoassay (EIA). On day 7, the mouse displaying the highest anti-E1E2 titer was sacrificed and a cell suspension of the spleen was prepared that was then mixed with K6H5/B5 heteromyeloma cells (kindly provided by Dr. Kris Thielemans, Free University of Brussels (VUB), Brussels, Belgium) at a 4:1 ratio. Polyethylene glycol 1500 (50% v/v; Boehringer Mannheim, Mannheim, Germany) was added for 2 min and then washed away. Fused cells (5x10^4^) were cultured in microtiter plates in 200 μL of medium supplemented with human recombinant insulin (10 μg/mL, Boehringer Mannheim), ouabain (1 μM, Sigma, St. Louis, MO), hypoxanthine-aminopterin-thymidine (Life Technologies, Belgium) and 10% v/v BM Condimed HI (Boehringer Mannheim). Cultures were replenished with fresh medium every other day and individual wells were checked for cell growth first and anti-E1E2 IgG production subsequently. Eight anti-E1E2 IgG-producing cultures were selected, subcloned several times and further expanded. After initial screening for reactivity against the HCV E1E2 protein (EIA), the neutralizing potential of the supernatant of all growing cultures was tested with neutralization assays using HCV pseudoparticles (HCVpp) of gt1a (isolate H77c). The human mAb with the strongest neutralizing capacity was selected and designated 2A5. Hybridoma cells producing mAb were propagated in a two-compartment bioreactor (Integra) and the antibody-containing culture supernatant was changed weekly. mAb 2A5 was purified using a protein G column (GE Healthcare Life Sciences) and concentrated using Amicon Centrifugal filters (Merck Millipore). The mAb 2A5 content of this preparation was determined using a human IgG ELISA Quantitation Set (Bethyl Laboratories).

**GNA capture, denaturation and competition EIA**

The binding affinity of mAb 2A5 was tested using enzyme immunoassay (EIA) with cell lysates containing recombinant HCV E1E2 glycoproteins. Expression vectors (pcDNA 3.1/Hygro^(+)^; Invitrogen) encoding E1E2 of different HCV isolates were used for transfection in 293T cells (ProFection mammalian transfection kit, Promega). Cell lysates containing E1E2 proteins were used for coating in EIA as previously described . Briefly, *Galanthus nivalis* agglutinin (GNA) coated EIA plates were blocked with blocking buffer (5% BSA in PBS) for 1 hour then loaded with cell lysates and incubated for 2 hours at room temperature (RT). After washing (PBS-0.05% Tween 20), two-fold serially diluted mAb (in antibody diluent: 5% BSA, 20% goat serum, 0.05% Tween 20 in PBS) was added and incubated for 90 minutes at RT. Detection was done using HRP-conjugated goat anti-human IgG (for 2A5, HC84.26, HC33.1) or goat anti-mouse IgG (for AP33). TMB (3,3’, 5,5’-Tetramethylbenidine) was added and the color intensity, corresponding to the concentration of bound antibody, was read at 450nm.

For denaturation EIA, cell lysates were incubated with 0.5% sodium dodecyl sulfate (SDS) and 5 mM dithiothreitol (DTT) for 15 min at 56°C and mAbs were used at 5 µg/mL. Competition EIA was performed using a previously described protocol with some modifications . Briefly, the GNA-coated plates were loaded with H77c E1E2 cell lysates diluted in blocking buffer (2.5% BSA, 2.5% goat serum, 0.1% Tween20 in PBS) and incubated for 2 hours at RT. Plates were washed three times with washing buffer (0.1% Tween20 in PBS) and 50 µl of serially diluted competing mAbs were added. After 1 hour, biotinylated-2A5 was added at a concentration corresponding to 60-75% of the maximum OD value and incubated for 1 hour at RT. Plates were washed 5 times and HRP-conjugated streptavidin was added for 30 min. After adding the TMB substrate, the OD values were measured.

**Production and entry inhibition of HCVpp**

HCV pseudoparticles covering genotypes 1-6 were produced as previously described . Briefly, 293T cells were co-transfected with two expression vectors containing either the full length E1E2 of HCV, or an HIV-I Gag-Pol packaging construct together with a luciferase reporter. Supernatant containing HCVpp was collected 48 hours post transfection and the infectivity was tested by titration on Hep3B cells. For the HCVpp neutralization assays, serial dilutions of mAbs 2A5 or AP33 were mixed with HCVpp and incubated at 37°C for 1 hour before addition to Hep3B cells. After 72 hours, the luciferase activity corresponding to the degree of infection was quantified using a luciferase quantification kit (Promega). The 50% inhibitory concentration (IC_50_; the mAb concentration achieving 50% inhibition of infection) was determined using a non-linear regression analysis (GraphPad Prism 6 Software).

**HCVcc production and neutralization assay**

HCVcc, representing genotypes 1-7, were produced as previously described . Briefly, XbaI (NEB) linearized HCV encoding plasmids were used for *in vitro* transcription using T7 RNA polymerase (Promega). We used a full-length gt1a HCV isolate (TNcc) and several JFH1-based chimeras: H77c (gt1a), J4 (gt1b), JC1 (J6/JFH1; gt2a), S52 (gt3a), UKN3A1.28c (gt3a), UKN3A13.15 (gt3a), ED43 (gt4a), SA13 (gt5a), HK6a (gt6a), QC69 (gt7a) . For virion production, Huh-7.5-RFP cells were transfected with 10 µg of HCV RNA using Lipofectamine 2000 reagent (Invitrogen). The culture supernatant, collected from day 2 to 7, was pooled, sterile filtered and stored at –80°C in small aliquots. For HCVcc neutralization assays, 1.3x10^4^ Huh7.5RFP cells were seeded in 96 well plates and incubated at 37°C. The next day, three-fold serial dilutions of mAbs 2A5 or AP33 (starting at 90 µg/mL) were incubated with 50-100 number of focus forming units (FFU) of HCVcc (37°C for 1 hour). Next, the virus-antibody mixture or the virus only was added to the cells and incubated for 4 hours. After washing, cells were incubated for another 48 hours. After fixation with 4% paraformaldehyde, cells were permeabilized with Triton X100 and immunostained with an anti-NS5A mAb (clone 9E10, kindly provided by Dr. Charles M. Rice, Rockefeller University, NYC, USA) in combination with an Alexa 647-conjugated goat anti-mouse IgG (Invitrogen). For analyses with the mouse mAb AP33, sera of HCV antibody positive patients and Alexa 647 conjugated goat anti-human IgG were used as a primary and secondary antibody, respectively. Similar concentrations of the irrelevant mAb Mal1C (raised against malaria) were used as negative control. The number of FFU was determined with the BD Pathway 435 High Content Bioimager (BD Biosciences, Erembodegem, Belgium). The 50% inhibitory concentration (IC_50_; the mAb concentration achieving 50% inhibition of infection) was estimated using a non-linear regression analysis (Graphpad Prism 6 Software).

***In vivo* HCV challenge**

Human liver chimeric mice were produced as previously described . Briefly, two weeks after birth, homozygous urokinase-type plasminogen activator-severe combined immunodeficiency (uPA^+/+^-SCID) mice were transplanted by intra-splenic injection with 10^6^ cryopreserved primary human hepatocytes. All mice received hepatocytes from the same donor (HH223; BD Biosciences, Belgium). Several weeks after transplantation, mice were bled to quantify human albumin plasma levels by EIA (Bethyl Laboratories). Mice with human albumin levels ranging from 2.9 to 8 mg/mL were used in the study. In passive immunization studies, mice were intraperitoneally injected with 1 mg of mAb 2A5 three days before challenge with a 100% infectious dose of gt1a (mH77; 10^4^ IU), gt1b (mP05; 10^4^ IU), gt4a (mED43; 10^4^ IU) or gt6a (mHK6a; 10^5^ IU) . The viral isolates H77, ED43 and HK6a were originally isolated from the acute phase of experimentally infected chimpanzees (kindly provided by Dr. Robert H. Purcell and Dr. Jens Bukh, NIAID, NIH, USA) . Viral isolate P05 was originally isolated from a chronically infected liver-transplanted HCV patient . The patient virus was injected into humanized mice to generate new viral stocks and eliminate anti-HCV antibodies. The prefix m refers to mouse-passaged virus. To avoid the use of a sub-optimal infectious dose in the *in vivo* prevention studies, the 100% infectious dose was estimated by titrating the viral isolates in humanized mice. After challenge, mice were bled weekly and HCV-RNA levels in mouse plasma were quantified using the COBAS Ampliprep/COBAS TaqMan HCV test (Roche Diagnostics). The limit of quantification (LOQ) of the assay is 15 IU/mL. Since mouse plasma was tested at a dilution of 1/50, the LOQ increases to 750 IU/mL. The mAb 2A5 levels in mice plasma were quantified using a human IgG quantification ELISA kit (Bethyl Laboratories). The Animal Ethics Committee of the Faculty of Medicine and Health Sciences of the Ghent University approved the *in vivo* study protocol.

**Amplification of the HCV envelope region**

The E1E2-region of virions derived from control as well as non-protected mice was amplified using RT-PCR. Briefly, after viral RNA extraction from 50 µl plasma (ZR Viral RNA kit, Zymo Research), cDNA synthesis was performed using superscript III reverse transcriptase (Invitrogen) and random primers. Next, full-length E1E2 was amplified by nested PCR. The first PCR was done using LongAmp DNA polymerase (NEB) and specific primers: 5` CGT AGG TCG CGT AAC TTG GGT AA 3′ and 5` GTG CGC CTC GGC CCT GGT GAT AAA 3` for genotype 1b (P05); 5' CCC GGA ATT TGG GTA AGG TC 3' and 5' AAG GCA CCT TGA GCA AAC TG 3' for genotype 6a (HK6a). For the second PCR, we used Pfu DNA polymerase (Promega) and specific primers: 5' TAT AGA TAT CAT GGG GTA CAT TCC GCT CGT C 3' and 5' ATA TGA TAT CTT ACT CAG CCT GAG CTA TCA G3' for P05; 5' CTA CTC TCG TGC CTC ACA AC 3' and 5' GCC GCA TTG AGG ACG ACA AG 3' for HK6a. Full length E1E2 was cloned into the Zero Blunt vector (Invitrogen) and the insert was sequenced (GATC Biotech, Germany). Sequences were multiple aligned and analyzed using BioEdit software version 7.2.0, Clone manager 9 professional and CLC main workbench version 7.6.4 (QIAGEN).

**Ethics statement**

The mouse experiments described in this manuscript were approved by the Animal Ethics Committee of the Faculty of Medicine and Health Sciences of the Ghent University (identification number: ECD 10/35). All mouse experiments were conducted according to European (Directive 2010/63/EU) and Belgian (Law of December 27, 2012 and Royal Decree of May 29, 2013) legislation. Collection of patient samples at Ghent University was approved by the Ghent University Hospital Ethical Review Committee (reference numbers 94/137, 99/02 and 2005/119) and after written informed consent of the donor. Human material including serum from patients with chronic HCV infection and followed at the Strasbourg University Hospitals, Strasbourg, France was obtained with written informed consent from all subjects (Institutional Review Board # CPP 10-17). HCVpp generated at the University of Nottingham originate from patient samples from the Trent Hepatitis C cohort, which has been described elsewhere . Briefly, patients who attended one of the study centers were asked to consent to inclusion. Data on demography, risk factors for infection, dates of exposure to risk, laboratory investigations, biopsies and treatments are stored in a linked anonymized database. HCV-infected patients with hemophilia or HIV co-infection and those who were identified through screening on renal dialysis units were identifiable within the database but excluded from further analysis, as the natural history of disease may differ in these groups. Ethics approval for the Trent HCV cohort and associated studies was obtained from the Yorkshire Multicenter Research Ethics Committee.

**Statistical analysis**

Statistical significance was calculated by Wilcoxon’s matched-pairs signed-ranks test using GraphPad Prism software version 6. P-values below 0.05 were considered statistically significant.

**References**

Bukh, J., Meuleman, P., Tellier, R., Engle, R.E., Feinstone, S.M., Eder, G., Satterfield, W.C., Govindarajan, S., Krawczynski, K., Miller, R.H., Leroux-Roels, G., Purcell, R.H., 2010. Challenge Pools of Hepatitis C Virus Genotypes 1-6 Prototype Strains: Replication Fitness and Pathogenicity in Chimpanzees and Human Liver-Chimeric Mouse Models. J Infect Dis 201, 1381-1389.

Depraetere, S., Verhoye, L., Leclercq, G., Leroux-Roels, G., 2001. Human B cell growth and differentiation in the spleen of immunodeficient mice. Journal of immunology 166, 2929-2936.

Desombere, I., Fafi-Kremer, S., Van Houtte, F., Pessaux, P., Farhoudi, A., Heydmann, L., Verhoye, L., Cole, S., McKeating, J.A., Leroux-Roels, G., Baumert, T.F., Patel, A.H., Meuleman, P., 2016. Monoclonal anti-envelope antibody AP33 protects humanized mice against a patient-derived hepatitis C virus challenge. Hepatology 63, 1120-1134.

Dubuisson, J., Hsu, H.H., Cheung, R.C., Greenberg, H.B., Russell, D.G., Rice, C.M., 1994. Formation and intracellular localization of hepatitis C virus envelope glycoprotein complexes expressed by recombinant vaccinia and Sindbis viruses. Journal of virology 68, 6147-6160.

Fafi-Kremer, S., Fofana, I., Soulier, E., Carolla, P., Meuleman, P., Leroux-Roels, G., Patel, A.H., Cosset, F.L., Pessaux, P., Doffoel, M., Wolf, P., Stoll-Keller, F., Baumert, T.F., 2010. Viral entry and escape from antibody-mediated neutralization influence hepatitis C virus reinfection in liver transplantation. J Exp Med 207, 2019-2031.

Foquet, L., Hermsen, C.C., van Gemert, G.J., Van Braeckel, E., Weening, K.E., Sauerwein, R., Meuleman, P., Leroux-Roels, G., 2014. Vaccine-induced monoclonal antibodies targeting circumsporozoite protein prevent Plasmodium falciparum infection. J Clin Invest 124, 140-144.

Gottwein, J.M., Scheel, T.K., Hoegh, A.M., Lademann, J.B., Eugen-Olsen, J., Lisby, G., Bukh, J., 2007. Robust hepatitis C genotype 3a cell culture releasing adapted intergenotypic 3a/2a (S52/JFH1) viruses. Gastroenterology 133, 1614-1626.

Gottwein, J.M., Scheel, T.K., Jensen, T.B., Lademann, J.B., Prentoe, J.C., Knudsen, M.L., Hoegh, A.M., Bukh, J., 2009. Development and characterization of hepatitis C virus genotype 1-7 cell culture systems: role of CD81 and scavenger receptor class B type I and effect of antiviral drugs. Hepatology 49, 364-377.

Hsu, M., Zhang, J., Flint, M., Logvinoff, C., Cheng-Mayer, C., Rice, C.M., McKeating, J.A., 2003. Hepatitis C virus glycoproteins mediate pH-dependent cell entry of pseudotyped retroviral particles. P Natl Acad Sci USA 100, 7271-7276.

Jensen, T.B., Gottwein, J.M., Scheel, T.K., Hoegh, A.M., Eugen-Olsen, J., Bukh, J., 2008. Highly efficient JFH1-based cell-culture system for hepatitis C virus genotype 5a: failure of homologous neutralizing-antibody treatment to control infection. J Infect Dis 198, 1756-1765.

Johansson, D.X., Voisset, C., Tarr, A.W., Aung, M., Ball, J.K., Dubuisson, J., Persson, M.A.A., 2007. Human combinatorial libraries yield rare antibodies that broadly neutralize hepatitis C virus. P Natl Acad Sci USA 104, 16269-16274.

Keck, Z., Wang, W.Y., Wang, Y., Lau, P., Carlsen, T.H.R., Prentoe, J., Xia, J.M., Patel, A.H., Bukh, J., Foung, S.K.H., 2013. Cooperativity in Virus Neutralization by Human Monoclonal Antibodies to Two Adjacent Regions Located at the Amino Terminus of Hepatitis C Virus E2 Glycoprotein. Journal of virology 87, 37-51.

Keck, Z.Y., Xia, J., Cai, Z., Li, T.K., Owsianka, A.M., Patel, A.H., Luo, G., Foung, S.K., 2007. Immunogenic and functional organization of hepatitis C virus (HCV) glycoprotein E2 on infectious HCV virions. Journal of virology 81, 1043-1047.

Keck, Z.Y., Xia, J., Wang, Y., Wang, W., Krey, T., Prentoe, J., Carlsen, T., Li, A.Y., Patel, A.H., Lemon, S.M., Bukh, J., Rey, F.A., Foung, S.K., 2012. Human monoclonal antibodies to a novel cluster of conformational epitopes on HCV E2 with resistance to neutralization escape in a genotype 2a isolate. Plos Pathog 8, e1002653.

Li, Y.P., Ramirez, S., Jensen, S.B., Purcell, R.H., Gottwein, J.M., Bukh, J., 2012. Highly efficient full-length hepatitis C virus genotype 1 (strain TN) infectious culture system. Proc Natl Acad Sci U S A 109, 19757-19762.

Mercer, D.F., Schiller, D.E., Elliott, J.F., Douglas, D.N., Hao, C.H., Rinfret, A., Addison, W.R., Fischer, K.P., Churchill, T.A., Lakey, J.R.T., Tyrrell, D.L.J., Kneteman, N.M., 2001. Hepatitis C virus replication in mice with chimeric human livers. Nat Med 7, 927-933.

Meuleman, P., Bukh, J., Verhoye, L., Farhoudi, A., Vanwolleghem, T., Wang, R.Y., Desombere, I., Alter, H., Purcell, R.H., Leroux-Roels, G., 2011. In Vivo Evaluation of the Cross-Genotype Neutralizing Activity of Polyclonal Antibodies Against Hepatitis C Virus. Hepatology 53, 755-762.

Meuleman, P., Libbrecht, L., De Vos, R., de Hemptinne, B., Gevaert, K., Vandekerckhove, J., Roskams, T., Leroux-Roels, G., 2005. Morphological and biochemical characterization of a human liver in a uPA-SCID mouse chimera. Hepatology 41, 847-856.

Meuleman, P., Vanlandschoot, P., Leroux-Roels, G., 2003. A simple and rapid method to determine the zygosity of uPA-transgenic SCID mice. Biochem Bioph Res Co 308, 375-378.

Neal, K.R., Trent Hepatitis, C.S.G., Ramsay, S., Thomson, B.J., Irving, W.L., 2007. Excess mortality rates in a cohort of patients infected with the hepatitis C virus: a prospective study. Gut 56, 1098-1104.

Owsianka, A., Tarr, A.W., Juttla, V.S., Lavillette, D., Bartosch, B., Cosset, F.L., Ball, J.K., Patel, A.H., 2005. Monoclonal antibody AP33 defines a broadly neutralizing epitope on the hepatitis C virus E2 envelope glycoprotein. Journal of virology 79, 11095-11104.

Potter, J.A., Owsianka, A.M., Jeffery, N., Matthews, D.J., Keck, Z.Y., Lau, P., Foung, S.K.H., Taylor, G.L., Patel, A.H., 2012. Toward a Hepatitis C Virus Vaccine: the Structural Basis of Hepatitis C Virus Neutralization by AP33, a Broadly Neutralizing Antibody. Journal of virology 86, 12923-12932.

Scheel, T.K., Gottwein, J.M., Jensen, T.B., Prentoe, J.C., Hoegh, A.M., Alter, H.J., Eugen-Olsen, J., Bukh, J., 2008. Development of JFH1-based cell culture systems for hepatitis C virus genotype 4a and evidence for cross-genotype neutralization. Proc Natl Acad Sci U S A 105, 997-1002.

Tarr, A.W., Owsianka, A.M., Timms, J.M., McClure, C.P., Brown, R.J.P., Hickling, T.P., Pietschmann, T., Bartenschlager, R., Patel, A.H., Ball, J.K., 2006. Characterization of the hepatitis C virus E2 epitope defined by the broadly neutralizing monoclonal antibody AP33. Hepatology 43, 592-601.

Tarr, A.W., Urbanowicz, R.A., Hamed, M.R., Albecka, A., McClure, C.P., Brown, R.J., Irving, W.L., Dubuisson, J., Ball, J.K., 2011. Hepatitis C patient-derived glycoproteins exhibit marked differences in susceptibility to serum neutralizing antibodies: genetic subtype defines antigenic but not neutralization serotype. Journal of virology 85, 4246-4257.

Urbanowicz, R.A., McClure, C.P., Brown, R.J., Tsoleridis, T., Persson, M.A., Krey, T., Irving, W.L., Ball, J.K., Tarr, A.W., 2015. A Diverse Panel of Hepatitis C Virus Glycoproteins for Use in Vaccine Research Reveals Extremes of Monoclonal Antibody Neutralization Resistance. Journal of virology 90, 3288-3301.

Wakita, T., Pietschmann, T., Kato, T., Date, T., Miyamoto, M., Zhao, Z.J., Murthy, K., Habermann, A., Krausslich, H.G., Mizokami, M., Bartenschlager, R., Liang, T.J., 2005. Production of infectious hepatitis C virus in tissue culture from a cloned viral genome. Nat Med 11, 791-796.

Wang, Y., Keck, Z.Y., Saha, A., Xia, J., Conrad, F., Lou, J., Eckart, M., Marks, J.D., Foung, S.K., 2011. Affinity maturation to improve human monoclonal antibody neutralization potency and breadth against hepatitis C virus. The Journal of biological chemistry 286, 44218-44233.

**Supporting information**

**Fig. S1. Neutralization of gt3a HCVcc by mAbs 2A5.** HCVcc expressing the structural proteins of gt3a isolates UKN3a1.28c and UKN3A13.15 were pre-incubated for 1 hour at 37°C with three-fold serial dilutions of mAb 2A5. After pre-incubation, the mixture was transferred to Huh7.5.RFP cells and HCV-infected foci were visualized 2 days later using an NS5A-specific antibody. Results are expressed as percentage of HCVcc infectivity (mean +/- standard deviation (error bars))**.** All conditions were performed in triplicate.

**Fig. S2. Epitope mapping of mAb 2A5 scanning the complete E1E2 sequence.** A custom-made chip with printed, 14-AA overlapping 15-mer peptides spanning the complete E1E2-sequence peptides was incubated with mAb 2A5. Bound antibodies were visualized with a fluorescently-labeled secondary antibody. Positive spots indicate antibody-reactive peptides and allow identification of the epitope (A). Peptide sequences comprising the region AA429-448 stained positive (mid-right and lower left) (A and B). Positive spots in the outer rim are used as a control and for localization purposes. The peptide sequences and chip layout can be found in supplementary File S1.

**Table S1: Neutralization activity of mAb 2A5 against HCV pseudoparticles derived from clinical isolates.**

**Table S2: Overview of mice HCV RNA and human IgG levels.**

**Table S1: Neutralization activity of mAb 2A5 against HCV pseudoparticles derived from clinical isolates.**

| **Isolate** | **Genotype** | **Accession no.** | **IC_50_ (µg/mL)** |
| --- | --- | --- | --- |
|  |  |  | **mAb 2A5** |
| **UKN1A20.8** | 1a | EU155192 | 7.57 |
| **UKN1B5.23** | 1b | AY734976 | 0.57 |
| **UKN2A1.2** | 2a | AY734977 | 0.47 |
| **J6** | 2a | AF177036 | 77.38 |
| **UKN2B2.8** | 2b | AY734983 | 8.78 |
| **UKN3A13.6** | 3a | AY894683 | 0.35 |
| **UKN4.11.1** | 4 | AY734986 | 38.16 |
| **UKN5.15.7** | 5 | EF427672 | 0.02 |
| **UKN6.5.8** | 6 | EF427671 | 0.34 |
