# Supplementary material for: A novel neutralizing human monoclonal antibody broadly abrogates hepatitis C virus infection in vitro and in vivo
Source: Antiviral Res. Author manuscript; Available in PMC 2018 Jan 25. (PMC5785094; doi:10.1016/j.antiviral.2017.10.015)
Supplement: Supplementary file 2 [file NIHMS935524-supplement-3.docx]

**Table S2: Overview of mice HCV RNA and human IgG levels.**

|  |  |  | **Day -3** |  |  | **HCV RNA (IU/ml)** |  |  |  |  |  |
| --- | --- | --- | --- | --- | --- | --- | --- | --- | --- | --- | --- |
| **HCV strain** | **Dose (IU/mouse)** | **Mouse ID** | **HuAlb (mg/ml)** | **Antibody** | **Plamsa IgG Week 1 (µg/ml)** | **Week 1** | **Week 2** | **Week 3** | **Week 4** | **Week 6** | **Week 8** |
|  |  |  |  |  |  |  |  |  |  |  |  |
| **mH77C** | 10^4^ | B1096L | 7.6 | - | **-** | 2.1x10^6^ | 1.4x10^6^ | ND | 1.2x10^6^ | 9.3x10^5^ | 5.9x10^5^ |
|  | 10^4^ | B1093 | 3.8 | - | **-** | 1.7x10^4^ | 1.4x10^5^ | ND | 2.1x10^6^ | animal died |  |
|  | 10^4^ | K1738R | 6.4 | - | **-** | 3.9x10^3^ | 8.10x10^5^ | animal died |  |  |  |
|  |  |  |  |  |  |  |  |  |  |  |  |
|  | 10^4^ | K1727 | 7.8 | 2A5 | **77.7** | <7.5x10^2^ | <7.5x10^2^ | ND | <7.5x10^2^ | <7.5x10^2^ | <7.5x10^2^ |
|  | 10^4^ | K1755R | 5.6 | 2A5 | **171.0** | <7.5x10^2^ | <7.5x10^2^ | ND | <7.5x10^2^ | <7.5x10^2^ | <7.5x10^2^ |
|  | 10^4^ | K1731RL | 3.6 | 2A5 | **111.0** | <7.5x10^2^ | <7.5x10^2^ | ND | <7.5x10^2^ | <7.5x10^2^ | <7.5x10^2^ |
|  | 10^4^ | B1073 | 3 | 2A5 | **92.0** | <7.5x10^2^ | <7.5x10^2^ | <7.5x10^2^ | <7.5x10^2^ | animal died |  |
|  |  |  |  |  |  |  |  |  |  |  |  |
| **mP05** | 10^4^ | K1115R | 5.5 | - | **-** | 3.2x10^5^ | 5.4x10^5^ | 1.4x10^6^ | 9.2x10^5^ | ND | ND |
|  | 10^4^ | B313R | 4.7 | - | **-** | 1.5x10^4^ | 4.9x10^5^ | 6.5x10^6^ | 4.8x10^5^ | ND | ND |
|  | 10^4^ | B311 | 6.4 | - | **-** | 1.7x10^5^ | 6.1x10^5^ | 1.3x10^6^ | 7.3x10^5^ | ND | 8.4x10^5^ |
|  | 10^4^ | B311R | 4.6 | - | **-** | 1.8x10^3^ | 2.4x10^6^ | animal died |  |  |  |
|  |  |  |  |  |  |  |  |  |  |  |  |
|  | 10^4^ | B553RL | 4.6 | 2A5 | **110.0** | <7.5x10^2^ | <7.5x10^2^ | 1.1x10^5^ | ND | ND | 3.4x10^5^ |
|  | 10^4^ | B586RL | 3.8 | 2A5 | **42.0** | 2.8x10^3^ | 3.6x10^3^ | 2.8x10^7^ | ND | ND | 3.7x10^5^ |
|  | 10^4^ | K1217L | 4.1 | 2A5 | **202.0** | <7.5x10^2^ | <7.5x10^2^ | ND | <7.5x10^2^ | ND | <7.5x10^2^ |
|  | 10^4^ | K1208LL | 4.2 | 2A5 | **75.0** | 3.3x10^4^ | 2.1x10^7^ | ND | 1.2x10^7^ | ND | 3.5x10^5^ |
|  | 10^4^ | B432R | 5.7 | 2A5 | **47.5** | <7.5x10^2^ | <7.5x10^2^ | ND | <7.5x10^2^ | ND | <7.5x10^2^ |
|  | 10^4^ | B449L | 4.3 | 2A5 | **84.0** | <7.5x10^2^ | 1.5x10^5^ | ND | 1.6x10^6^ | ND | 1.8x10^6^ |
|  |  |  |  |  |  |  |  |  |  |  |  |
| **mED43** | 10^4^ | B449L | 7 | - | **-** | 2.6x10^3^ | 3.1x10^5^ | ND | 1.8x10^5^ | 1.1x10^5^ | animal died |
|  |  |  |  |  |  |  |  |  |  |  |  |
|  | 10^4^ | K1329 | 3.6 | 2A5 | **130.0** | <7.5x10^2^ | <7.5x10^2^ | ND | <7.5x10^2^ | ND | <7.5x10^2^ |
|  | 10^4^ | K1329L | 6.6 | 2A5 | **44.5** | <7.5x10^2^ | <7.5x10^2^ | ND | <7.5x10^2^ | ND | <7.5x10^2^ |
|  | 10^4^ | K1329RL | 5.9 | 2A5 | **86.0** | <7.5x10^2^ | <7.5x10^2^ | ND | <7.5x10^2^ | ND | <7.5x10^2^ |
|  | 10^4^ | B533RL | 8 | 2A5 | **40.0** | <7.5x10^2^ | <7.5x10^2^ | ND | <7.5x10^2^ | ND | <7.5x10^2^ |
|  |  |  |  |  |  |  |  |  |  |  |  |
| **mHK6a** | 10^5^ | B519 | 2.9 | - | **-** | 1.6x10^3^ | 4.2x10^4^ | ND | 1.2x10^6^ | ND | 8.2x10^5^ |
|  | 10^5^ | B519L | 6 | - | **-** | 4.8x10^3^ | 6.0x10^4^ | ND | 7.9x10^4^ | ND | 4.5x10^4^ |
|  |  |  |  |  |  |  |  |  |  |  |  |
|  | 10^5^ | B465 | 5.8 | 2A5 | **65.0** | <7.5x10^2^ | <7.5x10^2^ | ND | <7.5x10^2^ | ND | <7.5x10^2^ |
|  | 10^5^ | K1315R | 6.1 | 2A5 | **25.5** | <7.5x10^2^ | <7.5x10^2^ | 6.4x10^3^ | 5.3x10^4^ | ND | ND |
|  | 10^5^ | K1315L | 4 | 2A5 | **23.0** | <7.5x10^2^ | <7.5x10^2^ | ND | <7.5x10^2^ | ND | <7.5x10^2^ |
|  |  |  |  |  |  |  |  |  |  |  |  |
| ND: Not Determined |  |  |  |  |  |  |  |  |  |  |  |
